# Puerperal Eclampsia

**Published:** 1869-07

**Authors:** C. C. F. Gay


BUFFALO

### Medical and Surgical Journal.

VOL. VIII. JULY, 1869. No. 12.

### Original Communications.

ART. I.—*Puerperal Eclampsia.* By C. C. F. Gay, M. D.

The true pathology of these convulsions is, at this present moment, and doubtless will for all time to come, remain somewhat obscure. He who reads the writings of our immediate predecessors along with those of our cotemporaries, will be forcibly impressed with the fact that between the writings of the former and those of the latter, there is very great contrariety of views expressed, both in regard to the etiology and pathology of this disease. The cause of these convulsions is pretty well defined and understood by writers and practitioners of our own time. A reference to the views of authors upon the causation of convulsions will, I trust, not be considered out of place by those who may do me the honor to read this paper.

M. Chailly claims that pregnancy is one of the essential causes of convulsions, and included in the category of predisposing causes, he mentions a lymphatic temperament, and particularly infiltration, and under the head of occasional causes, "lively and sudden moral impressions, irritation, and an acute pain." "But," he continues, "the most evident occasional cause, is the sympathetic reaction of the uterus on the economy." Pregnancy, which Chailly regards as an essential cause I should rather be inclined to regard not as cause, but as a condition precedent, merely.

Ramsbotham divides the causes into proximate and remote: under the former he places "pressure on the brain," but adds that the "disease has proved fatal often without any organic lesion being evident on dissection, or without even the vessels being observed to be preternaturally full." Under the head of remote causes he says, "it is not my wish to enter at any length, because the subject is at best, but unsatisfactory, and little understood."

Gooch merely and briefly makes allusion to the preternatural susceptibility of the nervous system and plethora as causes, and thence proceeds to describe the phenomena of convulsions.

Meigs, writing in 1850, says: "there are many circumstances, the concurrence of which tends to the development of the eclampsic convulsions of pregnant, puerperal and lying-in women. For many women, the whole state of gestation, from conception to labor, is a state of nervous excitement or hyperæsthesia, which renders the subject specially obnoxious, under the application of exciting causes, to convulsive or irregular non-conformable innervation." Two years later Meigs writes and putting the subject matter in an interrogative form says: "Can this excess of propensity to eclampsia in primipara be attributed to any other cause than those excessive sanguine determinations to the head above indicated?"

In these brief quotations I have limited myself to these distinguished authors, omitting not inadvertently but purposely, quotations of the earlier as well as more recent authors and writers. After this cursory glance at the causation, I will pass to the pathology of puerperal convulsions. If in this regard our knowledge is limited, it is because of the fact that autopsies have failed to impart as much light and knowledge as could be desired and hoped for. Should I ask: "What is a puerperal convulsion?" I should have much more difficulty in obtaining a satisfactory and intelligent response, than were I to ask, what are the symptoms or phenomena that attend upon a convulsion. Is a puerperal convulsion epilepsy or apoplexy? or, is it neither the one nor the other? Is it a disease *sui generis*, unlike in all essential elements, that of any other disease, and does it have a pathology peculiarly its own, and one dissimilar to any and every other malady? These are questions which come directly in the way and cross one's path when prosecuting inquires about, and attempting to arrive at, a knowledge of the true pathology of these anomalous convulsions. I say anomalous, because I believe these convulsions may be shown to be unlike those of any other. If puerperal convulsions are neither epileptic nor apoplectic, then must we look elsewhere than to these diseases for the veritable pathology.

It is a singular fact that these several authors whom I have quoted, although their writings are of comparatively recent date, make no allusion to that morbific condition which is now universally accepted as the cause and pathology of these convulsions. It is true that two of these authors make mention of *infiltration*, but are everywhere silent as to the condition of the urinary secretion, of albuminuria, or of Bright's disease of the kidneys, or of their congestion.

Meigs believes that pressure of the gravid uterus upon the aorta and cava, lays the foundation of all subsequent trouble, that "these restraints to the downward circulation tend to hyperæmia of the brain." The same pressure, he says, interrupts the return of the venous blood from the extremities, and hence when that pressure reaches its maximum the feet and legs become œdematous or anasarcous. His theory is based upon mechanical causes alone, and there is no essential difference between his theory and that of the other authors whom I have quoted. In the view of all of them the brain is the objective point; it is the secondary or primary lesion of the brain which puts out the life of the woman, in their view, and yet they acknowledge that dissection may reveal nothing that would lead one to suppose the brain had the least instrumentality in causing fatal result, there being no morbid condition of that organ discoverable.

The views of these several authors being incorrect and their positions untenable, it follows as a logical sequence that their heroic measures of depletion by large and oft-repeated venesection could not be sustained by a constantly inquiring and laborious profession. That Meigs was "well up" in a knowledge of the science and the art of medicine at his age and time of writing, no one will doubt, I think. At the time he wrote, he had no equal as a teacher and writer in this country, and I believe no superior in any country. It is because of my great admiration of him as a man and as a teacher that I have taken the liberty of quoting from him more fully than from any other author. Had he written twenty years later his expressed views upon the pathology of convulsions would have been in accordance, I have no doubt, with the now commonly accepted views of the entire medical profession. Critics have from time to time been rather severe with him, but compared with Meigs these critics are as rush-lights before the full blaze of the sun at meridian.

It is not my purpose to make quotations from authors of our time, but merely to give a summary of the views of present writers upon the pathology of convulsions. One purpose of quoting authors belonging to the generation just past, has been, to exhibit the contrast between the state of medical knowledge then and now, that it may serve as an incentive to activity and labor, that one may be restrained from the rest that comes from conviction, that there is nothing more to learn, that perfection has been reached, and that there can exist no possibility or probability that our successors by their advances in the knowledge of the truth, will be able as easily to set aside our views and crude theories as we are those who have immediately preceded us.

It appears to us most singular that renal disease, either structural or functional, giving rise to albuminuria should have so long escaped the observations of men, but such is the fact. There may be something yet remaining, some fact bearing upon the subject of the pathology of convulsions escaping the observation of the present generation of physicians. The field, entire, of uræmic poisoning, has not yet perhaps been surveyed. One fact is established, and that is, that urea is possessed of toxicological properties, that it is an irritant when retained in the blood. Experiments demonstrate that when injected into the veins of animals, urea causes convulsions, proving clearly that these convulsions are the result of non-elimination of the toxic element of the blood called urea, the kidneys having failed to eliminate it. Whether this failure depends upon renal congestion, caused by pressure of a gravid uterus upon the large vessels, according to Meigs, or renal congestion caused by some other agency, or whether the renal disease is a primary affection or structural disease, I can have nothing new to offer; at all events, we know that when this toxic element is present, whatever its cause, or when by applying the simple test, either of acid or heat, the urine is found loaded with albumen, convulsions may be looked for with as much certainty of their advent as we may look forward for the development of any fact, principle, or law, which science has established.

With these remarks I close the subject of the pathology of puerperal eclampsia, conscious as I am that nothing new has been stated, or attempted to be uttered, that the readers of this article have not had knowledge of from actual personal observations or reading.

A few words now upon the diagnosis and prognosis of this disease, after which I will venture to submit some views upon what I regard the best plan of managing this frightful accident or complication of the puerperal state.

Diagnosis would seem to be so easily made that it may appear supererogatory to say much about it, but I have some thoughts which I wish to submit for consideration, which may possibly be considered of some value to such of my readers as are not hypercritical. It is very likely to be found true that puerperal eclampsia may have been sometimes applied to convulsive disease as a misnomer; convulsions occurring during the puerperal state need not of necessity be puerperal convulsions. A fever during the puerperal state need not of necessity be a puerperal fever, for then any intercurrent disease, as erysipelas or rubeola, etc., might *a priori* lay claim to the prefix puerperal, and we should have puerperal erysipelas and puerperal rubeola. Puerperal convulsions are not of frequent occurrence, neither does apoplexy or epilepsy often occur. There is reason for the supposition, I think, that an occasional case occurs during the puerperal state of some other form of convulsions than that usually called puerperal, and this other form of convulsions may arise from epilepsy or from any cause which would produce cerebral congestion, effusion or extravasation, and therefore in this way the true character of the disease which I am considering may have been obscured and confounded with that of some other form of convulsion, which in all essential characteristics is non-puerperal.

The differential diagnosis between these three forms of disease to which I have alluded, is not difficult; in epilepsy and apoplexy we should look only for a single convulsion, while in true puerperal convulsions we should expect many consecutive convulsions. But during the puerperal state the cerebral vessels may have, from one cause or another, attained to such a condition of hyperæsthesia as to give rise to consecutive convulsions, and assimilate so nearly those from uræmic poisoning as that the one condition might be mistaken for the other, and that we should fail to resort to venesection in the one case, when it alone could save life, or resort to it in the other when its employment might compromise the patient's life. It becomes of the first importance then when called to the bed-side of a woman laboring under convulsions while in the puerperal state, to form correct diagnosis, and strive to satisfy our minds that the convulsions we witness might or might not have occurred from causes entirely independent of and foreign to the puerperal condition of the woman. Convulsions occur before, during and after labor. The relative frequency of these three several periods is stated by Ramsbotham, in 59 cases which occurred under his observation to be, 14 occurring before labor was instituted, 28 during the process, and 14 after its termination. Convulsions may be postponed after labor until ten, twelve or fourteen days. This fact, viz.: the occurrence of convulsions after labor has been terminated, teaches me that delivery is not a remedy for convulsions occurring in the first two of the three periods above named.

It is of interest to know the relative frequency of convulsions in any given number of labors and the relative mortality attending them. Dr. Collins reports that in 16,414 labors under his care, there were 30 cases of puerperal convulsions, of which there were 25 recoveries and 5 deaths, or one case of convulsions in every 547 cases of the disease. This is probably a near approach to accuracy. Madame Boivin reports that of 20,257 labors under her care in the Maternité at Paris, that 19 were affected with eclampsia, or 1 case in every 1,000 labors. Churchell collected tables of 96,903 labors, in which 159 cases of convulsions occurred, giving 1 in 609. Of the 30 cases given by Collins, 29 were primipara. Ramsbotham, Sr., records 22 cases, of which 15 cases were primipara. Madame Lachapelle estimates the mortality from this disease to be 50 per cent., but statistics show, I think, a much more favorable result.

*Preventive Treatment.*—Physicians, I have reason to believe, have not often times received credit when credit was due them for having arrested attacks of convulsions. Many more attacks have been prevented by timely aid and advice of the sagacious physician than have been cured. I feel confident that I have several times prevented these convulsions; at least I have had every reason for thus supposing. I have at least given timely attention and treatment to that abnormal condition made visible and apparent by œdema or anasarca previous to the completion of the full period of utero-gestation, and by dispelling the outward and visible signs, viz.: the œdema of the extremities; have been induced to believe that I had warded off what would have resulted in perhaps fatal convulsions. Although my belief may not be well founded, still my convictions will constrain me to pursue the plan of preventive treatment which I have thought to be of so much value.

A lady applied to me for advice in her eighth month of pregnancy; her limbs were enormously swollen, but no albumen was detected in the urine. She feared convulsions, as she had them in her first labor. It is true, as I have shown by statistics, that these convulsions occur most frequently in primipara, for this reason: neither the patient nor myself nor any one else could confidently expect that she would have them in her second confinement, although the same symptoms were present in both instances; besides this, neither the patient nor myself had cause, perhaps, to assert positively that the treatment by saline aperients and venesection warded off the anticipated attack of convulsions, although such was the belief. Treatment in one case might be denied any efficacy on account of the spontaneous subsidence of infiltration and œdema in the absence of any treatment whatever in another case, which not unfrequently occurs. Convulsions in a first labor do not by any means insure immunity from them in a second labor.

As negative evidence in support of the efficacy of preventive treatment, I may cite a case wherein my patient had convulsions in her first and second labors. Both labors were preceded and accompanied by very great anasarca, and no treatment had been instituted for its relief, or of the renal disease which was its cause. Had a physician been consulted in time, I am of opinion that he would by the use of venesection, saline aperients and diuretics, have prevented the advent of convulsions in these two labors.

Within a few weeks I was called to a primipara who was excessively anasarcous, the swelling extending up to and upon the face. She was within two weeks of the completion of the full term. I ordered a saline cathartic of sulphate magnesia ℥i every morning, and also the potassa nitras. This treatment was followed with the gradual but only partial subsidence of the bloating when she entered the lying-in hospital, and was attended by my friend, Dr. Potter, who informs me that she barely escaped convulsions. The preliminary or preventive treatment, was not begun early enough to positively insure immunity from attack, but sufficiently early, as the statement of Dr. Potter shows, to have brought the case within the pale of safety.

I have on record many cases showing the efficacy of timely medication in the prevention of convulsions, and believe general blood-letting should be resorted to oftener than it is, as it is said truthfully, an ounce of prevention is better than a ton of remedy. I should like, if my space permitted, to dwell somewhat more at length upon the importance and efficacy of preventive treatment in anticipating eclampsia.

In regard to curative treatment, I desire to quote paragraphs from two authors, which I regard of very great value, if they be remembered and heeded by obstetricians. Dr. Meigs says: "The woman ought to be delivered as soon as possible, because the child being taken away, one great cause of, and provocation to, the eclampsia will be removed along with it." By the words "soon as possible"—and this is, I regard, the most important declaration contained in Dr. Meigs' paragraph—"I mean as soon as it can possibly be done with propriety. Of that propriety the accoucheur must be the judge; yet no one can be held excusable who absolutely forces the os and cervix uteri."

Dr. Gooch penned a paragraph which I am inclined to like. He says: "Take care of the convulsions and leave the uterus to take care of itself."

It happened to occur to me in my early obstetrical practice to attend a primipara in severe puerperal eclampsia; her age was nineteen years, of previous good health; she had anasarca, and had consulted no physician. While lying upon the sofa she was suddenly seized with convulsions and fell upon the floor. I saw her soon thereafter, found her unconscious, and convulsions occurring at nearly regular intervals. She had twelve in all, extending over a period of three hours. She was delivered in her unconscious state of a living child by the natural and unaided efforts of nature twenty-four hours after the initial convulsion, on March 15, 1850, and had a good recovery. I bled this patient twice, but a small quantity of blood was abstracted at either bleeding, the patient seemingly being thrown into a convulsion at each stroke of the lancet, which made venesection difficult. Not to exceed eight ounces of blood was lost; chloroform was used very sparingly, not then knowing that it ever had been used in these convulsions. I think, perhaps, I may claim priority in its use, as I do not now remember a recorded *successful* case prior to this date, wherein this agent had been used by any member of the profession, although the honor or credit, if any honor and credit be attached to priority, in administration of this anæsthetic in this disease—has been claimed by others as early as 1848, or even before the discovery of its anæsthetic properties. But this is a digression for which I must ask pardon.

The opium treatment in this case of convulsions was somewhat heroic. Of course this case antedates the use of the hypodermic syringe many years, therefore opium had to be given by the stomach, if possible, to force it down, or given by rectum. One drachm of tr. opium was attempted to be given to my patient by the stomach, but some portion of it was lost. Soon thereafter two drachms were given by enema, and the same quantity repeated, as I hoped to control the convulsions by opium. The unconscious and comatose condition of my patient after cessation of convulsions led me to think, and a very able consulting physician shared my apprehensions, that I had fatally narcotized my patient. After twenty-four hours or more, consciousness gradually returned. Venesection, chloroform and opium with auxiliary measures as an active cathartic, sinapisms, etc., constituted the treatment.

I refer to this case occurring in my earlier practice with great satisfaction. The happy termination of it was very gratifying both to physician and friends. Since this case occurred I have ever been a fast friend to opium in puerperal convulsions. Dr. Ring will well remember my advocacy of it in a case I once saw with him, and more recently in another in which Dr. Wyckoff was associated with me, in which case I made free use of sul. morphia sub-cutaneously.

I do not believe the use of opium has yet been pushed to that extent its merit deserves; a woman in eclampsia will tolerate enormous doses of this narcotic, and indeed it is well known that the woman in disease from puerperal causes will usually be benefited, if ever benefited at all, just in proportion to the liberality with which this drug is administered. I believe that while convulsions last, morphia gr. i, is not too much to use hypodermically every half hour. The woman, or the brain of the woman, can tolerate this amount of opium better than she can tolerate convulsions. Too much efficacy has been accorded the use of the anæsthetic chloroform. It is a most valuable agent and indispensable in the treatment of convulsions. Its effect is speedy and powerful, but not permanent and abiding. It has been too exclusively relied upon, but as a remedy, if I were deprived of all others, and were obliged to use but one remedy, I should by all means choose this; but as there are other remedies at hand I would use chloroform as auxiliary and preliminary to any remedy known to be more permanent in its effect, and that remedy would be opium.

I should never think of excluding croton oil from the list of invaluable remedies. It can be more conveniently administered than any other cathartic. Two drops placed upon the tongue will be quite sure to operate speedily, and therefore as a remedy in these convulsions could not with safety be dispensed with.

I am not so much an advocate of general blood-letting as many are, but am of opinion that if blood had better be lost, that a sufficient quantity should be abstracted to make an impression upon the circulation, and resort to it as early as possible. I like it better as a preventive than as a curative agent. Should I treat another case of puerperal convulsions I should follow the advice of Meigs and Gooch, expressed in the two paragraphs above quoted. I should attend to the convulsions and let the uterus alone. I should not deliver my patient as soon as possible, unless the os uteri was fully dilated, and should not allow myself under any contingency to force an undilated os.

In writing this paper I have not sought to enlighten the older members of the profession. It has rather been my purpose to throw out some hints which may perchance serve as safe guidance to those who have but recently entered upon the responsible duties of a laborious and ever-harassing profession. It is my chief end and aim, in whatever I write, to do what I can and contribute what I can to the cultivation of the science and art of the profession of my choice; if I come short in this honest endeavor, it is not from want of some considerable thought and labor to which failure, in the consummation of such endeavor, can be attributed. He who writes what he expects others to do him the honor to read, becomes painfully conscious of his own imperfections, and these imperfections are fearfully enhanced when the vocation of the writer is such that the time usually devoted to sleep, or the snatches of time from professional and social engagements, are the only leisure moments allotted him. I must, therefore, ask indulgence of my readers, knowing full well that members of a liberal profession who can readily appreciate the difficulties attending the dual office of practitioner and writer, will be most willing to grant indulgence.

